# Microstructure and Mechanical Properties of 7072 Aluminum Alloy Joints Brazed Using (Ni, Y)–Modified Al–Si–Cu–Zn Filler Alloys

**DOI:** 10.3390/ma19010138

**Published:** 2025-12-31

**Authors:** Wei Guo, Ruihua Zhang, Zhen Xue, Hui Wang, Xinyu Zhang

**Affiliations:** 1 School of Mechanical Engineering, Nantong University, Nantong 226019, China; gwhy@163.com (W.G.); 13852807548@163.com (Z.X.); 2 School of Automotive and Traffic Engineering, Jiangsu University of Technology, Changzhou 213001, China; 19816153507@163.com; 3 Institute for Industrial Science, The University of Tokyo, Chiba 277-8574, Japan

**Keywords:** brazing, filler alloys, grain refinement, tensile strength, first principles

## Abstract

Aluminum–based brazing alloys have been developed for joining 7072 high–strength aluminum alloys. However, challenges related to their high melting points and joint softening still require further exploration. This study employs a combination of first–principles calculations and experimental techniques to examine the microstructure and mechanical properties of 7072 aluminum alloy joints brazed with (Ni, Y)–modified Al–Si–Cu–Zn filler alloys. Through the virtual crystal approximation (VCA) method, it was observed that the Al–10Si–10Cu–5Zn–*x*Ni–*y*Y (x = 0, 1.0, 2.0, 3.0, y = 0.2, 0.4, 0.6) filler alloy exhibits excellent mechanical stability, combining both high strength and reasonable ductility. Seven brazed joint samples with varying Ni and Y contents were fabricated using melting brazing and analyzed. The findings showed that Ni reduces the liquidus temperature of the filler, narrowing the melting range. This facilitates the conversion of the brittle Al_2_Cu phase into a more ductile Al_2_(Cu,Ni) phase, thus enhancing joint strength. Y acts as a heterogeneous nucleation site, promoting local undercooling, increasing the nucleation rate, and refining the microstructure. When the Ni content was 2.0 wt.% and the Y content was 0.4 wt.%, the tensile strength of the brazed joint reached a peak value of 295.1 MPa. Computational predictions align with the experimental results, confirming that first–principles calculations are a reliable method for predicting the properties of aluminum alloy brazing materials.

## 1. Introduction

The 7072 aluminum alloy, featuring high strength and outstanding corrosion resistance, is extensively applied in key structural parts such as frameworks, protective plates, and propulsion systems in manned spacecraft [1,2]. These applications make reliable joining technology a critical factor. In recent years, significant advancements have been made in the joining technology of aluminum alloys, including fusion welding [3,4], brazing [5,6] and fusion–brazing [7,8,9]. Fusion welding operations at temperatures exceeding the melting point of the base material, which can easily cause defects such as porosity and cracking, thereby affecting the integrity and quality of the weld. During brazing, the chemical interaction between the flux and base metal often leads to surface corrosion, which in turn degrades mechanical properties and reduces service life. Fusion–brazing, characterized by its lower joining temperature and flux–free process, offers a more efficient and dependable approach to aluminum alloy joining than conventional welding and brazing [10].

The performance of filler alloys is crucial for evaluating post–weld properties. The Zn–Al [11,12], Al–Cu [13] and Al–Si [14,15] series brazing alloys are commonly used for aluminum alloy brazing. Compared to Zn–Al and Al–Cu series alloys, Al–Si brazing alloys are more suitable for aluminum alloy components used in high–strength, high–temperature, and humid environments, as they exhibit superior wettability and excellent corrosion resistance [16]. However, Al–Si series brazing alloys are based on Al–12.6Si eutectic alloy, with a eutectic point of 577 °C. During the brazing process, this often leads to joint softening or even excessive melting of the base material. Therefore, it is imperative to develop high–strength, filler metals with low melting points are employed to create robust brazed joints in aluminum alloys.

Generally, incorporating alloying elements such as Ge, Zn, and Cu effectively lowers the melting point of Al–Si eutectic alloys. However, the needle–like Al–Ge phase tends to fragment the matrix, leading to poor workability and high production cost, which limits the commercial use of Al–Si–Ge fillers [17]. The addition of Zn reduces brittleness and enhances the ductility and toughness of brazed joints, though excessive Zn significantly deteriorates corrosion resistance. Muhamed N. M. et al. [18] reported that a moderate Zn addition in Al–Si–Zn fillers improved joint strength. In contrast, Cu provides a lower melting temperature, good processability, and high strength [19], making Al–Si–Cu fillers promising for aluminum brazing. Nevertheless, the generation of brittle Al–Cu intermetallic phases (IMCs) remains a major challenge. Abdulsalam M. et al. [20] found that excessive Al–Cu IMCs in brazed seams significantly reduced the joint strength when using Al–Si–Cu fillers.

To mitigate this issue, the introduction of alloying modifiers has been explored. Qin et al. [21] demonstrated that Ni addition effectively reduced brittleness and improved joint strength. Similarly, Gao et al. [22] conducted systematic studies on Ni–containing Al–Si–Cu fillers for 3003 aluminum alloys, establishing a correlation between microstructure and mechanical performance. Furthermore, rare–earth elements such as Ce, Y, La, and Yb have been shown to refine microstructures and enhance filler fluidity. Song X. C. et al. [23] reported that trace La and Yb additions refined grains within the brazing seam and increased joint tensile strength.

Despite extensive studies on Al–Si–Cu and Al–Si–Zn fillers, systematic investigations of multicomponent Al–Si–Cu–Zn–Ni–Y systems remain limited. In particular, the integration of first–principles calculations with experimental validation for predicting filler performance and microstructural evolution has yet to be fully developed. Moreover, the function of Zn within the Al–Si–Cu alloy matrix remains insufficiently clarified.

In this work, Al–10Si–10Cu–5Zn–*x*Ni–*y*Y fillers were designed and fabricated to reduce the melting temperature of the Al–12.6Si eutectic alloy and enhance joint strength. Cu was employed as a melting–point depressant, Zn as a ductility enhancer, Ni to compensate for the brittleness induced by Cu, and Y as a grain refiner. First–principles simulations were carried out to analyze the connection between the mechanical characteristics of the alloy and its Ni and Y constituents, followed by experimental investigations of their melting behavior, microstructure, and joint reliability, including tensile strength, interfacial morphology, and fracture characteristics.

Overall, while alloying and microalloying have been widely applied to optimize Al–Si–Cu filler performance, most existing studies focus on single–element systems or rely on empirical observations. A comprehensive understanding of multielement synergistic mechanisms—linking atomic–scale interactions to macroscopic joint performance—remains lacking.

To address this gap, the present study introduces Ni and rare–earth Y into the Al–10Si–10Cu–5Zn system to achieve synergistic modification. By combining first–principles calculations with fusion–brazing experiments, we systematically explored the impact of (Ni, Y) additions on the mechanical stability, melting characteristics, microstructural evolution, and joint strength of 7072 aluminum alloys.

The primary contributions of this work are summarized as follows:

(1) The Virtual Crystal Approximation (VCA) technique was employed to model the atomic–level mechanical properties of multicomponent filler alloys, enabling a computationally guided alloy design approach, which is validated through experimental results.
(2) The synergistic strengthening mechanism was clarified: Ni promotes the transformation of brittle Al_2_Cu into a networked Al_2_(Cu,Ni) phase, while Y induces heterogeneous nucleation and microstructural purification, resulting in grain refinement.
(3) An optimized filler composition was developed, achieving both a low liquidus temperature and high joint strength (295.1 MPa), significantly exceeding that of conventional Al–Si fillers.

This work deepens the understanding of the composition–microstructure–property relationship in multicomponent aluminum–based fillers and provides new theoretical insights and experimental evidence for the design of high–strength brazing materials with industrial applicability.

## 2. Materials and Methods

### 2.1. Computational Model Building

**Figure 1:**
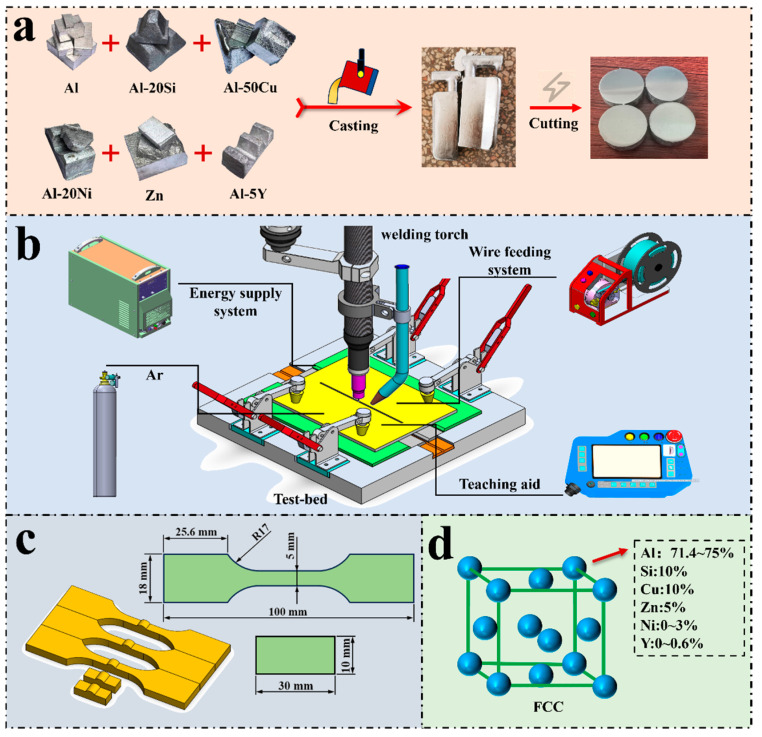
(**a**) Preparation process of the filler alloy; (**b**) Schematic diagram of fusion–brazing; (**c**) Samples for mechanical testing; (**d**) Schematic representation of the crystal structure of the Al–10Si–10Cu–5Zn–*x*Ni–*y*Y alloy.

In this study, first–principles simulations within the framework of density functional theory (DFT) were executed using the CASTEP module implemented in Materials Studio 2019 [24]. Using the CASTEP package 16.x within the Materials Studio platform, the virtual crystal approximation (VCA) method was applied to construct a face–centered cubic(FCC) structure model for the Al–10Si–10Cu–5Zn–*x*Ni–*y*Y multielement filler alloy [25], as shown in Figure 1d. The total electronic energy was computed using Vanderbilt’s norm–conserving pseudopotentials (NCPP) together with the Perdew–Burke–Ernzerhof (PBE) functional under the Generalized Gradient Approximation (GGA) framework [26]. Convergence tests for both cutoff energy and k–point sampling were performed using the Monkhorst–Pack scheme. The plane–wave basis was truncated at a cutoff energy of 900 eV, and a 16 × 16 × 16 k–point grid was adopted for Brillouin zone integration. During geometric optimization, convergence thresholds were defined such that the residual force on each atom was less than 0.05 eV/Å, the stress deviation did not exceed 0.1 GPa, the maximum atomic displacement was below 0.002 Å, and the total energy difference per atom was smaller than 2.0 × 10^−6^ eV/atom. These parameters ensured accurate relaxation of atomic configurations and lattice dimensions.

In this work, the virtual crystal approximation (VCA) is adopted as an efficient first–principles approach to model disordered multicomponent braze alloys by averaging the pseudopotentials of constituent elements, preserving the primitive cell periodicity and substantially reducing computational cost compared with explicit supercell models. VCA has been widely and successfully applied to predict elastic and mechanical trends in multicomponent systems, such as in Mg–Zn–Al–Sn alloys where computed elastic trends guide experimental validation [27] and in AlCoxCrFeNi high–entropy alloys where VCA enhances calculation efficiency while maintaining accuracy [28]. While VCA cannot capture local atomic disorder or short–range ordering, its predicted compositional trends in elastic properties remain valuable when correlated with microstructural and mechanical measurements.

### 2.2. Elastic Constants Analysis

The elastic constants of the multicomponent filler alloy were used to evaluate its mechanical response to external strain. According to reference [29], they are defined as follows:

(1) $$C_{\mathrm{ijkl}}={\left(\frac{\partial \sigma_{\mathrm{ij}(x)}}{\partial e_{\mathrm{kl}}}\right)}_{X}$$

In the equation, $\sigma_{ij}$ denotes the applied stress, $e_{kl}$ the strain, and X and x the coordinates before and after deformation, respectively. For cubic crystals, solving this equation provides three independent elastic constants: C_11_, C_12_, and C_44_. Additional parameters related to the elastic constants include the bulk modulus (B), Young’s modulus (E), shear modulus (G), and Poisson’s ratio (ν), as shown in the following formula [30].

(2) $$B=B_{V}=B_{R}=\left(C_{11}+{2C}_{12}\right)/3$$

(3) $$E=\frac{9BG}{3B+G}$$

(4) $$G_{V}=\left(C_{11}-C_{12}+{3C}_{44}\right)/5$$

(5) $$G_{R}=5\left(C_{11}-C_{12}\right)C_{44}/\left[4C_{44}+3\left(C_{11}-C_{12}\right)\right]$$

(6) $$G=\frac{G_{V}+G_{R}}{2}$$

(7) $$v=\frac{3B-E}{6B}$$

Due to the differing periodicity and density of atomic arrangements along various lattice directions in a crystal, the elastic modulus varies accordingly. Thus, anisotropy, as an important characteristic of crystals, is crucial for studying their elastic properties. The anisotropic behavior of filler alloys is usually expressed using the anisotropy index Au, as shown in the following formula [31]:

(8) $$A_{U}=5\frac{G_{V}}{G_{R}}+\frac{B_{V}}{B_{R}}-6$$

Additionally, the anisotropy in three dimensions was computed using the ElasticPOST 1.0.0 code.

## 3. Experimental Materials and Methods

The base material employed in this study was a 5 mm thick 7072 aluminum alloy plate, with its chemical composition provided in Table 1. The melting temperature of the alloy ranged from 580 to 610 °C, and its tensile strength at room temperature was approximately 500 MPa.

High–purity metals were used as raw materials, including Al (99.99 wt.%), Si (99.99 wt.%), Cu (99.99 wt.%), Zn (99.99 wt.%), Ni (99.9 wt.%), and Y (99.9 wt.%). The chemical compositions of the filler alloys were analyzed by inductively coupled plasma atomic emission spectroscopy (ICP–AES), and the deviations from the designed compositions were less than ±0.2 wt.%.

Intermediate alloys (Al–20Si, Al–50Cu, Al–20Ni, and Al–5Y) and high–purity Al and Zn were used as starting materials, with hexachloroethane serving as a refining agent. Melting was conducted in a well–type resistance furnace under a NaCl–KCl (1:1 by mass) molten–salt cover to produce a series of Al–Si–Cu–Zn–Ni and Al–Si–Cu–Zn–Ni–Y filler alloys, as listed in Table 2. To ensure compositional uniformity, each alloy was re–melted three times and annealed at 450 °C for 12 h. The preparation process is schematically illustrated in Figure 1a.

The melting and solidification behaviors of the filler alloys were investigated using a differential scanning calorimeter (DSC, Netzsch STA449F3, Netzsch Ger ä tebau GmbH, Selb, Germany) under high–purity nitrogen. The heating rate was 10 °C/min within a temperature range from room temperature to 600 °C. Temperature calibration was performed using indium and zinc standards (±0.5 °C accuracy).

The phase compositions were identified using an X–ray diffractometer (XRD, Rigaku, Tokyo, Japan, D/MAX–2500, Cu Kα, λ = 1.5406 Å) in the 2θ range of 20–90°, with a step size of 0.02° and a scanning rate of 2°/min. Each sample was measured three times to ensure reproducibility.

The microstructures of the filler alloys and brazed joints were analyzed using a field–emission scanning electron microscope (FE–SEM, ZEISS Gemini 500, Oberkochen, Germany) equipped with an energy–dispersive X–ray spectrometer (EDS, Oxford Instruments, Abingdon, UK). SEM imaging was conducted at 20 kV with a working distance of 8–10 mm and a beam current of 1.0 nA. Samples were mechanically polished and etched with Keller’s reagent (95 mL H_2_O + 2.5 mL HNO_3_ + 1.5 mL HCl + 1.0 mL HF). Consistent EDS conditions were applied to all samples to ensure comparability.

Brazing experiments were performed using an argon–protected arc as the heat source, as shown in Figure 1b. The base material (100 mm × 200 mm × 5 mm) and filler rod (3 mm × 3 mm × 300 mm) were joined in a butt configuration. Prior to brazing, both surfaces were ground with sandpaper, cleaned with ethanol, and dried. The brazing parameters were welding current 115 A, tungsten electrode height 10 mm, wire–feeding speed 220 mm/min, welding speed 3 mm/s, and argon flow rate 15 L/min.

Samples were prepared for microstructural observation and mechanical testing by wire electrical discharge machining (EDM), as illustrated in Figure 1c. The tensile strength of the brazed joints was measured using a universal testing machine, with each sample tested three times. The reported tensile strength values represent the averages of three tests.

The microhardness of the brazed joints was measured using a Vickers hardness tester (HV0.2) (Future–Tech Corp, Kawasaki–shi, Japan) with a 200 g load and a dwell time of 10 s. Ten indentation points were randomly selected on the polished cross–section of the brazed seam, avoiding grain boundaries and secondary phases. The mean value and standard deviation were calculated to assess data reliability.

## 4. Results and Discussions

### 4.1. Analysis of First Principles Results

#### 4.1.1. Mechanical Properties of the Al–10Si–10Cu–5Zn–*x*Ni–*y*Y Alloy

Elastic constants characterize the stress–strain relationship in a crystal lattice and reflect its mechanical behavior. To examine the mechanical properties of the Al–10Si–10Cu–5Zn–*x*Ni–*y*Y alloy, the elastic constants (C_11_, C_12_, and C_44_) were calculated using Materials Studio software. From these, the bulk modulus (B), Young’s modulus (E), and shear modulus (G) were derived. The results are presented in Table 3.

For cubic crystals, mechanical stability is defined by the conditions: C_11_ > 0, C_44_ > 0, C_11_ − C_12_ > 0, and C_11_ + 2C_12_ > 0. The calculated values confirm that the elastic constants of the Al–10Si–10Cu–5Zn–*x*Ni–*y*Y alloy satisfy these conditions, indicating its mechanical stability.

**Figure 2:**
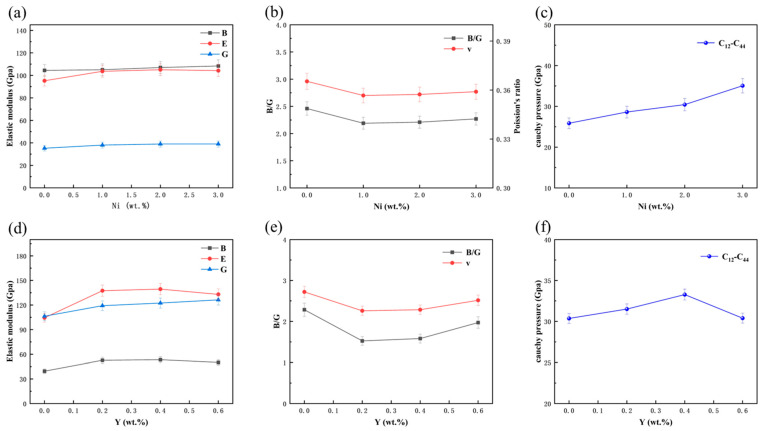
Mechanical properties of Al–10Si–10Cu–5Zn–*x*Ni–*y*Y alloys with varying Ni and Y content. (**a**,**d**) Elastic modulus; (**b**,**e**) Poisson’s ratio and B/G ratio; (**c**,**f**) Cauchy pressure.

Figure 2a,d illustrate the variations in bulk modulus, Young’s modulus, and shear modulus of the Al–10Si–10Cu–5Zn–*x*Ni–*y*Y alloy with different Ni and Y concentrations. As Ni and Y are added sequentially, all three moduli–B, E, and G–increase to varying extents, likely due to phase transformations induced by the inclusion of Ni and Y in the Al–10Si–10Cu–5Zn alloy. Additionally, the alloy’s bulk modulus increases with the rising content of Ni and Y. In contrast, the Young’s modulus and shear modulus initially increase and then decrease as the Ni and Y content increases. When the Ni content reaches 2.0 wt.% and Y content is 0.4 wt.%, the Young’s modulus and shear modulus increase sharply to 140.48 GPa and 53.64 GPa, respectively. Compared with the Al–10Si–10Cu–5Zn alloy, these values represent increases of 32.25% and 34.21%, respectively. This indicates that adding appropriate amounts of Ni and Y can enhance the alloy’s strength, hardness and brittleness.

Figure 2b,e present the bulk to shear modulus ratio (B/G) and Poisson’s ratio (ν) of the Al–10Si–10Cu–5Zn–*x*Ni–*y*Y alloy with different Ni and Y contents. According to Pugh’s criterion, materials with B/G ≥ 1.75 or ν ≥ 0.26 exhibit ductile behavior, while lower values indicate brittleness. As shown in Table 3 and Figure 2b–e, both B/G and ν of the alloy exceed these thresholds, suggesting good ductility. However, the introduction of Ni and Y decreases B/G and ν, indicating a ductile–to–brittle transition. With further increases in Ni and Y content, both parameters rise again, implying a partial recovery of ductility.

Figure 2c,f show the variation in Cauchy pressure (C_12_–C_44_–) for the Al–10Si–10Cu–5Zn–*x*Ni–*y*Y alloy with different Ni and Y content. Cauchy pressure is used to assess the strength and structural stability of materials. As seen in the figures, the Cauchy pressure of the alloy increases gradually with the addition of Ni and Y elements. When the Ni content reaches 2.0 wt.% and Y content 0.4 wt.%, the alloy’s Cauchy pressure reaches a maximum value of 33.29, an increase of 21.99% compared to the Al–10Si–10Cu–5Zn alloy. This indicates that Ni and Y elements can enhance the strength of the filler alloy. This is likely due to the grain refinement effect of Ni and Y elements in the filler alloy.

In summary, the addition of Ni and Y improves the strength and hardness of the Al–10Si–10Cu–5Zn–*x*Ni–*y*Y alloy, but also reduces its toughness, decreasing the alloy’s impact resistance and fracture toughness. Therefore, this trade–off must be carefully considered in material design.

**Figure 3:**
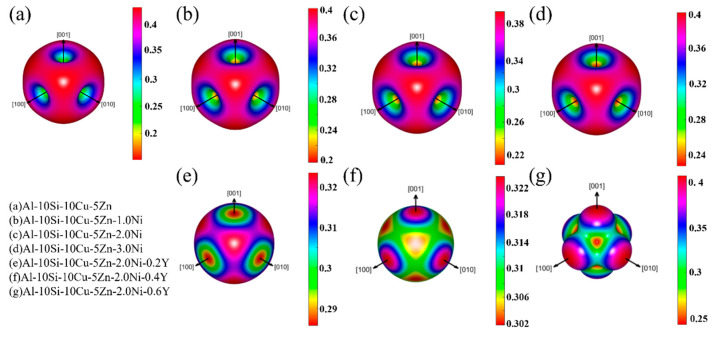
Three–dimensional Poisson’s ratio diagram of Al–10Si–10Cu–5Zn–*x*Ni–*y*Y alloy.

Additionally, to visually observe the effect of Ni and Y content on Al–10Si–10Cu–5Zn–*x*Ni–*y*Y alloys, Figure 3 depicts the 3D Poisson’s ratio of seven Al–10Si–10Cu–5Zn–*x*Ni–*y*Y fillers. According to the results calculated in Table 3, the Poisson’s ratio (ν of all alloys exceeds 0.25, indicating their ductility, which is consistent with the B/G ratio. Moreover, it is evident from the figure that Ni and Y elements reduce the Poisson’s ratio. Conversely, the Poisson’s ratio gradually increases with the addition of Ni and Y, suggesting that the crystal structure of the Al–10Si–10Cu–5Zn–*x*Ni–*y*Y alloys is transitioning from anisotropic to isotropic.

Based on the above analysis, Al–10Si–10Cu–5Zn–2Ni–0.4Y exhibits high strength and hardness (E = 140.48 GPa, G = 53.64 GPa), along with good toughness (Cauchy pressure = 33.29 GPa, v = 0.310) and isotropy in the (001), (100) and (010) directions, meeting the mechanical performance requirements of brazed joints.

#### 4.1.2. Electronic Structure Characterization of the Al–10Si–10Cu–5Zn–*x*Ni–*y*Y Alloy

**Figure 4:**
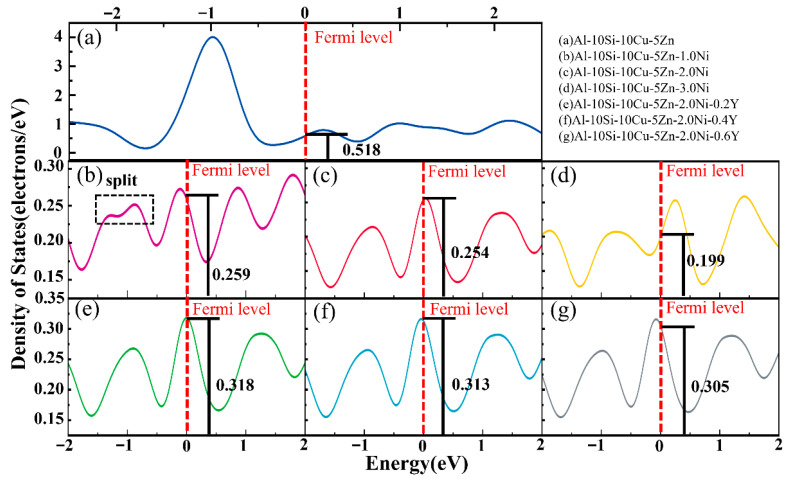
Electronic density of states analysis of Al–10Si–10Cu–5Zn–*x*Ni–*y*Y alloys.

To better understand how Ni and Y elements influence the mechanical behavior of Al–10Si–10Cu–5Zn alloys, the electronic density of states (DOS) of Al–10Si–10Cu–5Zn–xNi–yY alloys was analyzed, as shown in Figure 4. All compositions exhibit a non–zero DOS at the Fermi level, indicating their metallic nature.

With the addition of Ni and Y, the DOS at the Fermi level shows a gradual decrease. This trend suggests a possible enhancement of interatomic bonding strength and hence a qualitative tendency toward higher strength. However, such interpretation should be treated with caution, as the DOS–mechanical property relationship is not strictly quantitative, especially for multicomponent aluminum alloys.

The slight increase in DOS observed with Y addition may reflect a minor change in electronic structure, which could be related to its grain–refining effect observed experimentally. Moreover, when the Ni content reaches 1.0 wt.%, a splitting of the DOS peak is observed, implying that Ni may alter the electronic environment and potentially facilitate phase transformation in the Al–Si–Cu–Zn system.

Overall, the DOS analysis provides qualitative insight into the effect of Ni and Y on bonding characteristics, complementing the experimental observations of mechanical performance rather than quantitatively predicting them.

### 4.2. Filler Alloy

#### 4.2.1. Thermal Behavior of the Al–10Si–10Cu–5Zn–*x*Ni–*y*Y Alloy

**Figure 5:**
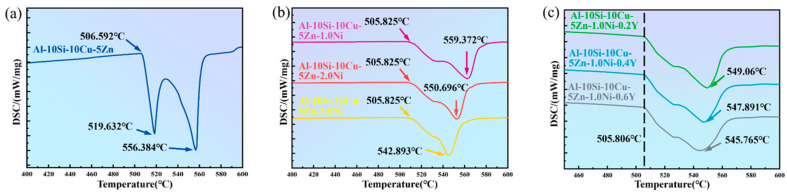
DSC melting characteristic curves of Al–10Si–10Cu–5Zn–*x*Ni–*y*Y alloys. (**a**) Al–10Si–10Cu–5Zn alloy without Ni or Y addition; (**b**) Al–10Si–10Cu–5Zn–xNi alloys with varying Ni content (x = 1.0–3.0 wt.%); (**c**) Al–10Si–10Cu–5Zn–1.0Ni–yY alloys with varying Y content (y = 0.2–0.6 wt.%).

Figure 5 illustrates the DSC melting curves of Al–10Si–10Cu–5Zn–*x*Ni–*y*Y alloys with varying Ni and Y contents. According to the DSC curves shown in the figure, the melting onset temperature (T_onset_), liquidus temperature (T_m_), melting termination temperature (T_offset_), and melting range (ΔT) of the filler alloys are summarized in Table 4. The analysis of the curves indicates that the DSC curves of the Al–10Si–10Cu–5Zn–*x*Ni–*y*Y filler alloys exhibit two endothermic peaks. Based on the Al–Si–Cu ternary phase diagram and the Al–Si–Cu–Ni quaternary phase diagram, these two endothermic peaks correspond to the Al–Si–Cu ternary eutectic point and the Al–Si–Cu–Ni quaternary eutectic point, respectively. This conclusion is consistent with the findings of previous researchers [32]. In particular, since the composition of the Al–10Si–10Cu–5Zn–xNi filler alloy is close to the eutectic point of the Al–Si–Cu–Ni quaternary alloy, its DSC curve remains relatively flat in the temperature range of 518–525 °C.

The incorporation of Ni generates new low–melting–point phases, which suppress the reverse reaction of α–Al and Si. Therefore, with the increasing addition of Ni, the solidus temperature of the Al–10Si–10Cu–5Zn–*x*Ni–*y*Y filled alloys remains relatively constant, while the liquidus temperature gradually decreases, and the melting range narrows, indicating that the filled alloys exhibit good brazing performance. Additionally, since the solubility of Y in Al is nearly zero (0.03%), it primarily exists in the form of alloy compounds. Consequently, the Y element has a minimal impact on the solid–liquid phase line temperature of the filled alloys, slightly reducing the liquidus temperature. When the Ni content remains constant, increasing the Y element content brings the two endothermic peaks closer together. This indicates that the rare earth element Y can enhance the stability of the characteristic transformation temperature of the filled alloys and improve their wettability.

In conclusion, adding Ni and Y elements reduces the melting range of Al–10Si–10Cu–5Zn–*x*Ni–*y*Y alloys. On the other hand, the Ni element can lower the liquidus temperature of the filled alloys, while the Y element can enhance their fluidity and wettability. This keeps the liquidus temperature consistently below 560 °C. Both factors are beneficial to the brazing process.

#### 4.2.2. Phase Composition of the Al–10Si–10Cu–5Zn–*x*Ni–*y*Y Alloy

**Figure 6:**
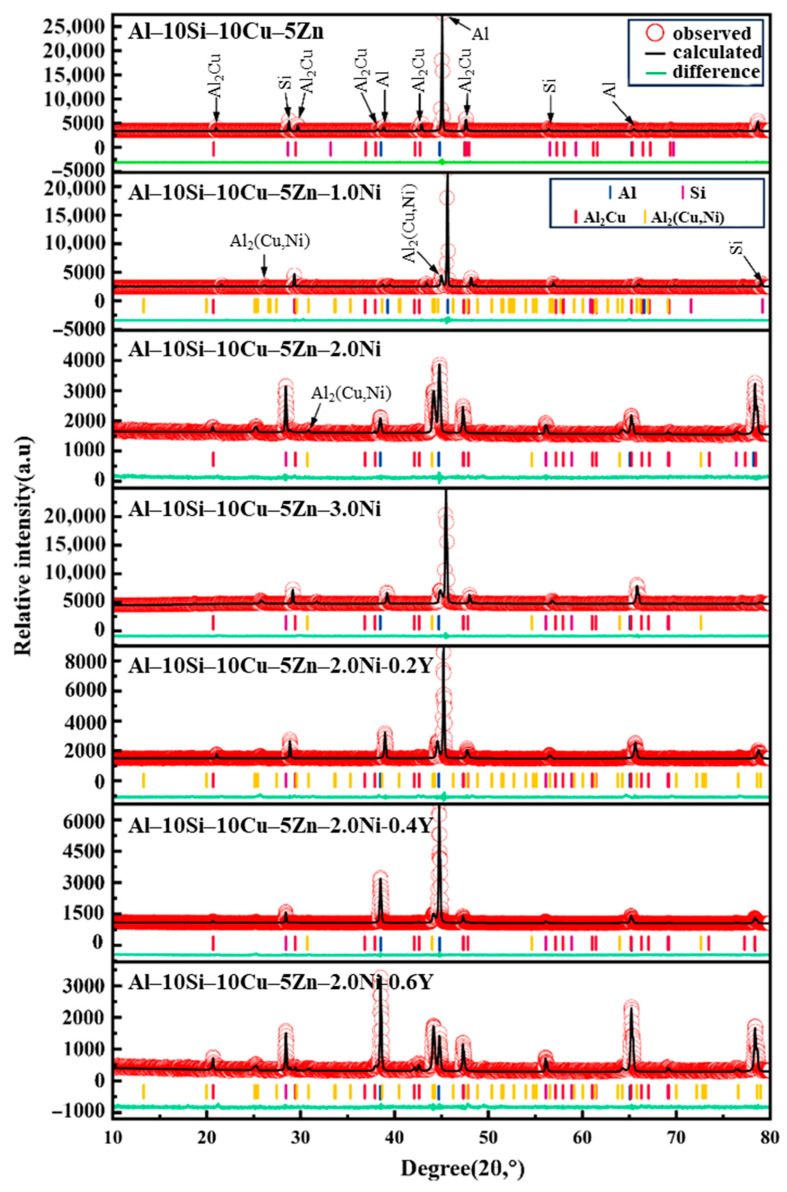
XRD patterns of Al–10Si–10Cu–5Zn–*x*Ni–*y*Y alloys.

Figure 6 presents the XRD patterns of Al–10Si–10Cu–5Zn–*x*Ni–*y*Y alloys with varying Ni and Y contents. From the figure, it can be observed that the Al–10Si–10Cu–5Zn filled alloy is primarily composed of α–Al phase, eutectic Si phase, and Al_2_Cu phase. After the addition of Ni, a series of new peaks appear in the XRD diffraction patterns, indicating that Ni can react with the Al_2_Cu phase to form the Al_2_(Cu,Ni) phase. Due to the low Y content, the XRD patterns do not show any elemental Y or Y–containing compound phases. Furthermore, Zn is highly soluble in Al, and the addition of a small amount of Zn does not alter the phase composition of the filled alloys. Therefore, no phase structures generated by Zn are observed in the XRD patterns.

By observing the variation of the peak intensity of the Al_2_(Cu,Ni) phase, it was found that with the increase in Ni content, the peak intensity initially decreases and then increases, indicating that an appropriate amount of Ni can reduce the grain size of the Al_2_(Cu,Ni) phase. Similarly, when the Ni content remains constant, the incorporation of Y introduces modifications to the filled alloys, resulting in a further reduction in the grain size of the phases. It is noteworthy that at a Y content of 0.6 wt.%, the crystalline plane related to the α–Al phase in the filled alloys undergoes a shift, which may be attributed to excessive rare earth Y leading to over–refinement of the grains. This affects the growth direction of the grains, thereby altering the arrangement of the crystal planes.

#### 4.2.3. Microstructure of the Al–10Si–10Cu–5Zn–*x*Ni–*y*Y Alloy

**Figure 7:**
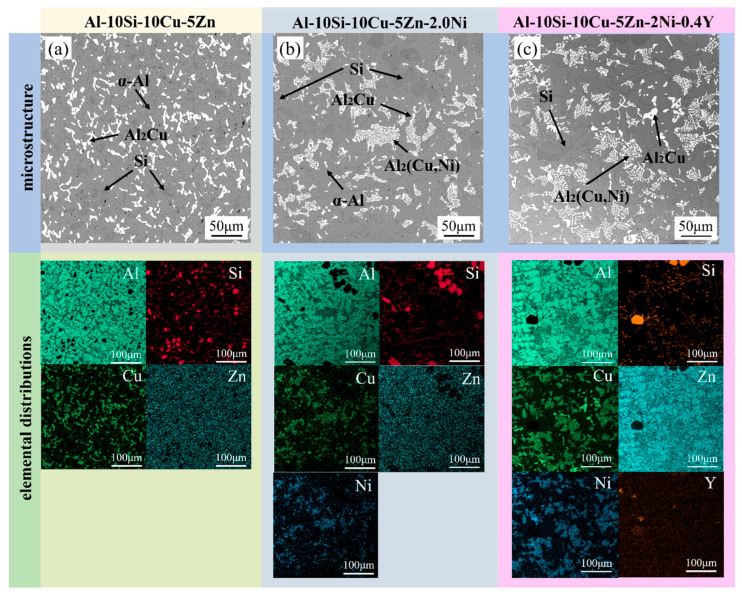
Microstructures and surface scans of Al–10Si–10Cu–5Zn, Al–10Si–10Cu–5Zn–2.0Ni, Al–10Si–10Cu–5Zn–2.0Ni–0.4Y filled alloys. (**a**) Al–10Si–10Cu–5Zn alloy showing α–Al, Si, and Al_2_Cu phases; (**b**) Al–10Si–10Cu–5Zn–2.0Ni alloy exhibiting α–Al, Si, Al_2_Cu, and Al_2_(Cu,Ni) phases; (**c**) Al–10Si–10Cu–5Zn–2.0Ni–0.4Y alloy containing α–Al, Si, Al_2_Cu, Al_2_(Cu,Ni), and refined structures with Y distribution.

Figure 7 displays the microstructural images and surface scan results of the Al–10Si–10Cu–5Zn, Al–10Si–10Cu–5Zn–2.0Ni and Al–10Si–10Cu–5Zn–2.0Ni–0.4Y filled alloys. It is clear that the Al–10Si–10Cu–5Zn filled alloy is primarily composed of gray α–Al phase, dark gray Si phase, and white Al_2_Cu phase. The Al–10Si–10Cu–5Zn–*x*Ni–*y*Y filled alloy consists of gray α–Al phase, dark gray Si phase, white Al_2_Cu phase and a networked Al_2_(Cu,Ni) phase. This indicates that under the influence of Ni, the Al_2_Cu phase in the filled alloys transforms into a mixture of Al2Cu phase and the networked white Al_2_(Cu,Ni) phase. This aligns with the findings derived from XRD analysis. The surface scan results also reveal that the distributions of Cu and Ni are highly overlapping, indicating the actual presence of the Al_2_(Cu,Ni) phase. Comparing Figure 7b,c, it is found that the microstructural structure of the filled alloys does not change with the addition of Y. However, the networked Al_2_(Cu,Ni) phase structure becomes more relaxed, which is attributed to rare earth Y reducing the driving force for the growth of Al_2_(Cu,Ni) and altering the growth morphology of intermetallic compounds, leading to a more dispersed Al_2_(Cu,Ni) structure in the filled alloys and reducing its cutting effect on the α–Al matrix. At the same time, the Si particles in the structure also decrease with the addition of Y. Furthermore, the Y element has a high chemical reactivity and reacts with hydrogen and oxide inclusions during the solidification process, generating hydrides and oxides that float to the surface, thereby reducing the porosity of the filled alloys (Figure 7c).

**Figure 8:**
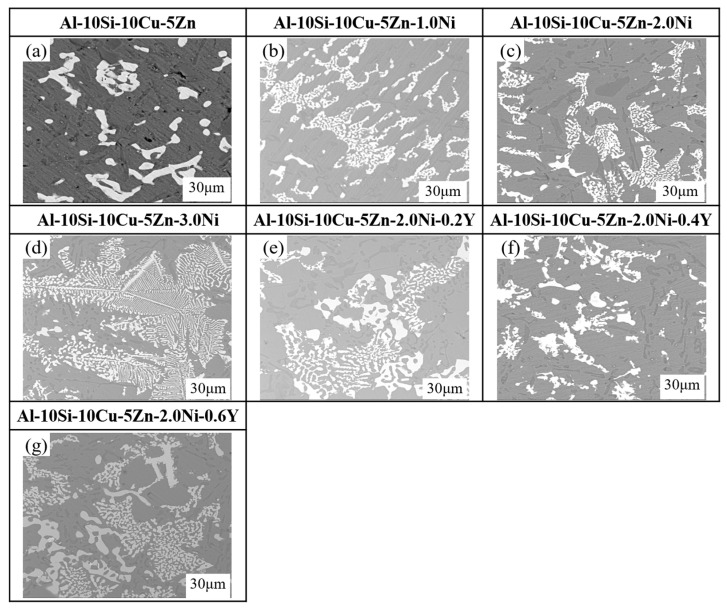
Microstructure of Al–10Si–10Cu–5Zn–*x*Ni–*y*Y filled alloys. (**a**) Al–10Si–10Cu–5Zn; (**b**) Al–10Si–10Cu–5Zn–1.0Ni; (**c**) Al–10Si–10Cu–5Zn–2.0Ni; (**d**) Al–10Si–10Cu–5Zn–3.0Ni; (**e**) Al–10Si–10Cu–5Zn–2.0Ni–0.2Y; (**f**) Al–10Si–10Cu–5Zn–2.0Ni–0.4Y; (**g**) Al–10Si–10Cu–5Zn–2.0Ni–0.6Y.

**Figure 9:**
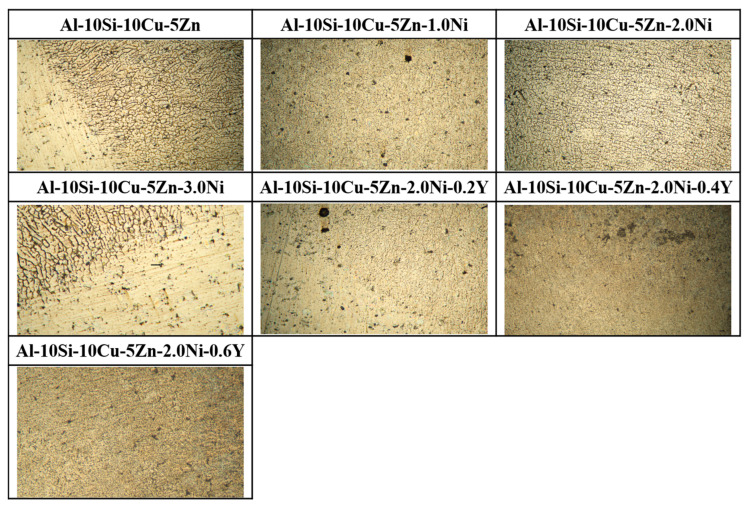
Metallographic microstructure of Al–10Si–10Cu–5Zn–*x*Ni–*y*Y filler alloys (100×).

Figure 8 displays the microstructural images of Al–10Si–10Cu–5Zn–*x*Ni–*y*Y alloys with varying Ni and Y contents. From Figure 8a–d, it can be observed that with the increase in Ni content, the quantity of the Al_2_Cu phase gradually decreases, while the quantity of the Al_2_(Cu,Ni) phase gradually increases. Simultaneously, the number of Si particles decreases, with some Si phases refining into a fibrous structure. From Figure 8e,f, it can be observed that with the increase in Y content, there is no significant change in the Si phase, while the grains of the Al_2_(Cu,Ni) phase and Al_2_Cu phase in the filled alloys become finer and exhibit a uniform dispersed distribution. Meanwhile, the metallographic examination of the Al–10Si–10Cu–5Zn–*x*Ni–*y*Y filler alloys was conducted to investigate the impact of Ni and Y additions on the microstructure. As shown in Figure 9, the microstructures at 100× magnification reveal significant differences in the morphology of the phases.

To describe the effects of Ni and Y on the grain changes in the microstructure of the filled alloys, the Image–Pro Plus 6.0 software was used to calculate the grain size and area fraction of the filled alloys. The grain size is defined as the equivalent circular diameter of the Al_2_Cu and Al_2_(Cu,Ni) phases. The results are summarized in Table 5. It is evident from the figure that after sequentially adding different contents of Ni and Y to the Al–10Si–10Cu–5Zn filled alloy, the grain size of the microstructure of the filled alloy gradually decreases. Based on the data in Table 5, it is concluded that as the Ni content increases, the average grain size of the filled alloys first decreases and then increases, reaching an average grain size of 3.82 μm at a Ni content of 2 wt.%, down from 5.21 μm. At the same time, the area fraction decreases from 10.3% to 7.9%. This indicates that an appropriate amount of Ni can reduce the grain size and area fraction of the Al_2_Cu and Al_2_(Cu,Ni) phases. Therefore, in this study, the Ni content in the filled alloys should not exceed 2.0 wt.%. Furthermore, when the Ni content remains constant, the addition of Y for modifying the filled alloys leads to observations of grain size changes. As the Y content increases, the average grain size and area fraction of the filled alloys gradually decrease. The average grain size decreases from 3.82 μm to 1.83 μm, while the area fraction decreases from 7.9% to 6.64%.

In summary, the refining effect of the metal element Ni on Al–Si–Cu–Zn alloys can be understood as follows: the addition of Ni transforms the brittle rod–like Al2Cu phase into a networked Al_2_(Cu,Ni) phase, hindering grain growth and thereby refining the grains. The refining effect of the rare earth element Y on Al–10Si–10Cu–5Zn–*x*Ni–*y*Y alloys can be understood as follows: the melting point of Y is significantly higher than those of the four elements Al, Si, Cu and Ni, allowing it to act as a center for heterogeneous nucleation. This increases the nucleation count per unit volume, resulting in a large number of undercooled regions, which increases the local undercooling and enhances the nucleation rate, thereby refining the grains.

### 4.3. Brazed Joint

#### 4.3.1. Microstructure of Brazed Joints

**Figure 10:**
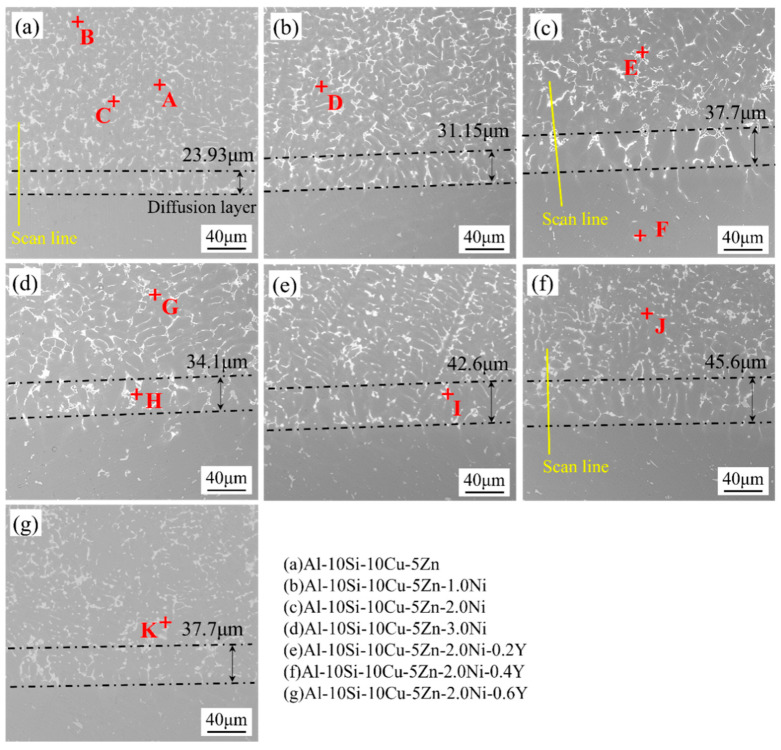
SEM cross–sectional microstructure of Al–10Si–10Cu–5Zn–*x*Ni–*y*Y brazing material brazed 7072 aluminum alloy joints.

Figure 10 illustrates the SEM cross–sectional morphology of the Al–10Si–10Cu–5Zn–*x*Ni–*y*Y filler alloy joined with 7072 aluminum alloy. The corresponding chemical compositions and phase identifications of the analyzed regions are given in Table 6. The interface appears compact and defect–free, indicating good metallurgical bonding. According to EDS results, the interfacial microstructure is mainly composed of α–Al, Si, Al_2_Cu, Al_2_(Cu,Ni), Al_6_(Mn,Fe)(F), and Al_6_(Mn,Fe)_2_Si(I) phases. The Mn and Fe elements, originating from the base metal, diffuse into the filler during brazing and combine with Al and Si to form Al_6_(Mn,Fe) and Al_6_(Mn,Fe)_2_Si phases.

Observation of the microstructure at the brazing interface reveals that large Si particles are not concentrated at the brazing interface, and the networked Al_2_(Cu,Ni) phase exhibits refined grain sizes, attributed to remelting of the filler and substrate during fusion–brazing. Increasing Ni content promotes the formation of the Al_2_(Cu,Ni) network and leads to the gradual shortening of needle–like Si phases, producing a uniform distribution of short–rod and fine particulate morphologies. This aligns perfectly with the analysis results depicted in Figure 8. After the addition of Y, the rod–like Si phase gradually disappears and transforms into fine particles, achieving a grain refinement effect. This reduces the sharp phase (Si)’s cracking effect on the brazed joint, thereby enhancing the joint’s performance. It is important to note that excessive Y can lead to over–modification; at the high temperatures of fusion–brazing, grain coarsening may occur (Figure 10g).

At the interface between the base material and the filler, lamellar or banded products were observed. Energy dispersive spectroscopy (EDS) analysis indicates that phase H corresponds to the Al_2_(Cu,Ni) phase. Considering the formation of a significant amount of Al_2_(Cu,Ni) intermetallic compounds (IMCs) at the interface between the base material and the filler alloy, it is believed that the bonding strength of the brazed joint is primarily determined by the thickness of the Al_2_(Cu,Ni) IMC layer. At the interface between the base material and the filler, as the Ni content increases from 0 wt.% to 2.0 wt.%, the thickness of the Al_2_(Cu,Ni) IMC layer increases from 23.93 μm to 45.6 μm. When the Ni content exceeds 2.0 wt.%, the thickness of the Al_2_(Cu,Ni) IMC layer decreases from 45.6 μm to 34.1 μm. This is related to the refining effect of the Ni element on the filler alloy’s microstructure. To enhance the refining effect on the Al_2_(Cu,Ni) phase, trace amounts of Y are added to the Al–10Si–10Cu–5Zn–2.0Ni filler alloy for modification treatment. It was found that as the Y content increases, the thickness of the IMC layer first increases and then decreases. The maximum thickness of 45.6 μm was achieved at a Y content of 0.4 wt.%. The results indicate that the addition of appropriate amounts of Ni and Y can effectively refine the grains in the brazed joint, thereby enhancing the performance of the brazed joint.

**Figure 11:**
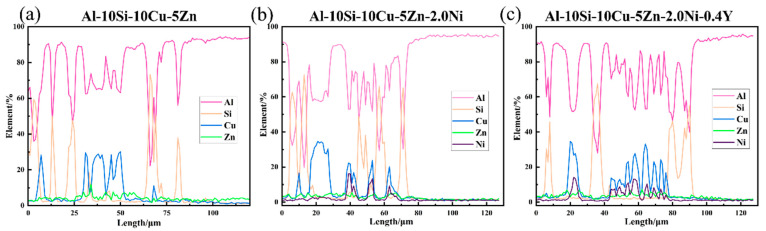
Elemental line scan distribution of Al–10Si–10Cu–5Zn, Al–10Si–10Cu–5Zn–2.0Ni, Al–10Si–10Cu–5Zn–2.0Ni–0.4Y brazing materials for brazing 7072 aluminum alloy.

Figure 11 shows the elemental line–scan distributions in panels (Figure 10a,c,f). The higher concentrations of Al, Si, Cu, and Ni at the brazed joint suggest the formation of Al_2_Cu, Al_2_(Cu,Ni), and Si phases. The Si phase typically exists in the form of Si particles or as Al–Si eutectics. By comparing the changes in peak widths after the addition of Ni and Y, it was found that both Ni and Y contribute to grain refinement, confirming the results presented in the figure. Additionally, due to its high solubility in Al, a small amount of Zn does not react with Al to form intermetallic compounds.

To further analyze the influence of different Ni and Y contents on the fracture behavior of brazed joints, semi quantitative statistics were conducted on the typical fracture morphology. By measuring the characteristic areas of dimples in high magnification SEM images, the average size and area ratio of dimples were estimated. The results showed that as the Ni content increased from 0 wt.% to 2.0 wt.%, the average diameter of the dimples increased from about 1.5 μm to 3.0 μm, and the area ratio increased from about 35% to 55%; After further adding 0.4 wt.% Y, the morphology of the tough dimples became more uniform and the distribution of tearing edges became denser, indicating that the fracture process was accompanied by stronger plastic deformation.

It should be pointed out that this statistical result is a semi quantitative estimation and only reflects the overall trend of fracture characteristics. Due to the unevenness of the fracture surface and the limitation of image resolution, there is a certain degree of error in the measurement of the size and proportion of the tough dimples. Therefore, the fracture analysis results of this study are mainly used to reveal the relative influence of Ni and Y on the fracture mechanism, rather than precise quantitative comparison.

#### 4.3.2. Mechanical Properties of Brazed Joints

**Figure 12:**
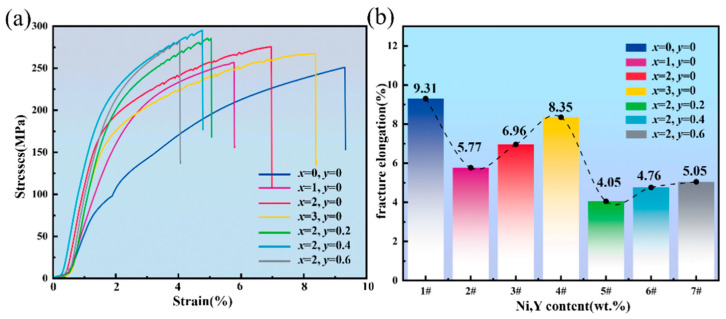
Mechanical properties of Al–10Si–10Cu–5Zn–*x*Ni–*y*Y brazed joints. (**a**) tensile strength; (**b**) elongation at break.

Figure 12a illustrates the tensile stress–strain behavior of Al–10Si–10Cu–5Zn–*x*Ni–*y*Y brazed joints with 7072 aluminum alloy, with detailed results summarized in Table 7. The curves display brittle fracture characteristics comprising elastic and strain–hardening regions. The tensile strength increases initially and then decreases with higher Ni content, reaching a peak value of 275.3 MPa at 2.0 wt.% Ni, corresponding to a 9.19% improvement over the Al–10Si–10Cu–5Zn joint.

The tensile strength, a key parameter for assessing brazed joint quality, was examined by introducing Y into the 2.0 wt.% Ni alloy. An increase followed by a decrease in tensile strength was observed as Y content rose, which agrees with the first–principles calculations. This indicates that trace amounts of Y beneficially modify the microstructure of the brazed joint, enhancing its strength. However, due to the limited solubility of Y in the brazing alloy, when the Y content is too high, excess Y elements can form a significant amount of precipitate phases, leading to a decrease in the joint’s tensile strength. At a Y content of 0.4 wt.%, the tensile strength reaches a maximum of 295.1 MPa, representing a 15.28% increase compared to the tensile strength of the Al–10Si–10Cu–5Zn brazed joint.

Figure 12b shows the trend of the elongation after fracture of the brazed joint. The data in the figure indicate that the elongation after fracture of the brazed joint significantly decreases with the successive addition of Ni and Y elements, leading to a reduction in the alloy’s plasticity. This indicates that as the tensile strength of the brazed joint increases, the fracture mode shifts from ductile fracture to brittle fracture. This is primarily related to the impediment of dislocation motion caused by Ni and Y [33]. Combining the data in the table, it is observed that the elongation after fracture rapidly decreases and then slowly increases with the gradual increase of Ni and Y content. At a tensile strength of 295.1 MPa for the brazed joint, the elongation after fracture is 4.76%.

**Figure 13:**
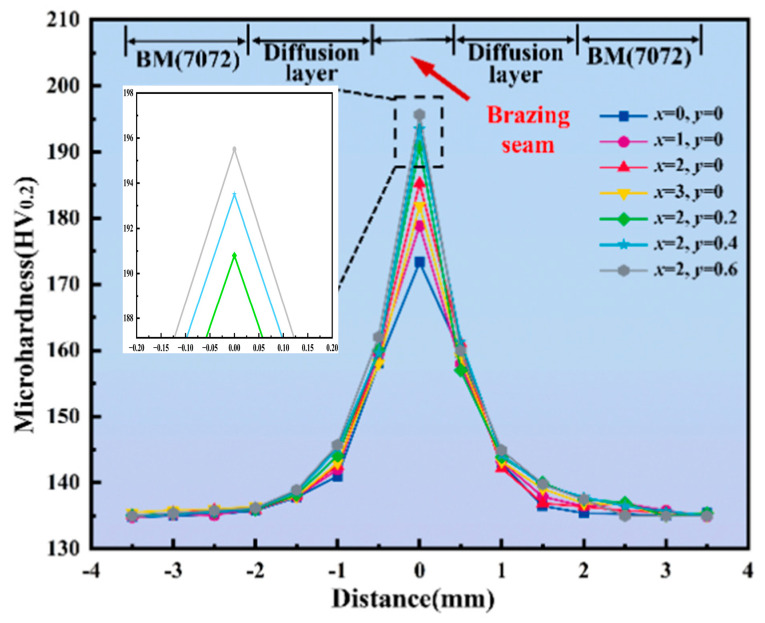
Hardness distribution curve of Al–10Si–10Cu–5Zn–*x*Ni–*y*Y brazed joints.

The degree of grain refinement in the structure of the brazed joint determines its hardness. Figure 13 illustrates the trend of the influence of Ni and Y content on the hardness of the brazed joint. The hardness variation trend represents a characteristic hardness curve of fusion–brazing. This is related to the thermal effects of arc brazing. Combining the data from Table 7, it can be observed that with the increase of Ni and Y content, the average hardness of the brazed joint shows an upward trend. This indicates that the elements Ni and Y have a favorable effect on grain refinement in the brazing alloy. This is consistent with the results of the Cauchy pressure (C_12_–C_44_) obtained from first–principles calculations. When the Ni content is 2.0 wt.% and the Y content is 0.6 wt.%, the average hardness of the brazed joint reaches a maximum value of 195.64 HV_0.2_.

A comparison indicates that the results of the mechanical property calculations based on first principles are consistent with the experimental results. Overall, Al–10Si–10Cu–5Zn–2.0Ni–0.4Y exhibits good plasticity and toughness (with a fracture elongation rate of 4.76%) while maintaining a high tensile strength of 295.1 MPa and a high hardness of 193.48 HV_0.2_. This material meets the mechanical performance requirements for brazed joints.

#### 4.3.3. Fracture Morphology of Brazed Joints

**Figure 14:**
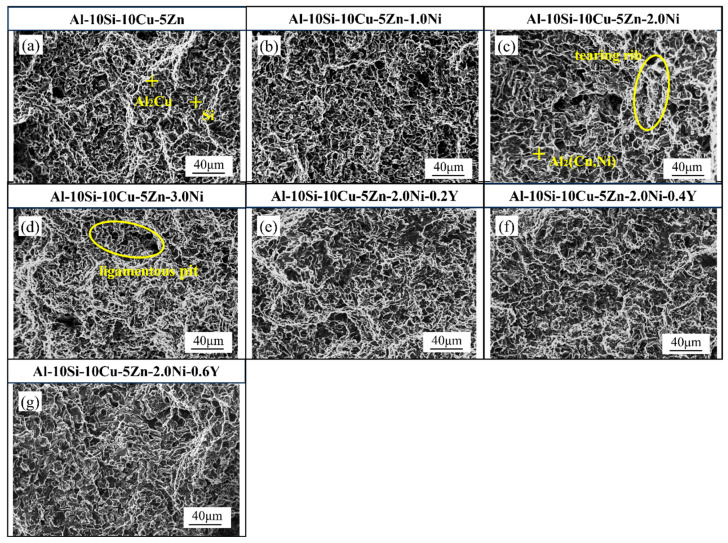
Fracture morphology of Al–10Si–10Cu–5Zn–*x*Ni–*y*Y brazed joints.

The fracture morphology of the brazed joint is a primary basis for analyzing its mechanical properties. Figure 14 shows the fracture morphology of the brazed joint. The fracture occurs in the diffusion zone between the base material and the filler alloy, where the fracture morphology is relatively flat, and plastic deformation is not significant. Additionally, a large number of dimples and tear ridges are observed, indicating that the fracture mode is a mixed–type fracture.

Table 8 presents the EDS analysis results of the typical phases of the fracture morphology for the Al–10Si–10Cu–5Zn–*x*Ni–*y*Y brazed joint. The results indicate that phases A, B and C correspond to the Si phase, Al_2_Cu phase, and Al_2_(Cu,Ni) phase, respectively. During the brazing process, mutual diffusion occurs between the elements of the base material and the molten filler alloy, leading to the formation of a sound metallurgical bond at their interface.

As the Ni content increases, the number of tear ridges increases, the size of the dimples gradually enlarges, and the fracture morphology changes from a fine and uniform equiaxed structure to an irregular undulation characterized by large dimples and dense tear ridges (Figure 14b–d). The formation of large dimples facilitates an increase in elongation, while the fracture mode transitions from ductile fracture to brittle fracture. Analysis of the microstructure and fracture morphology of the brazed joint reveals a significant presence of Al_2_(Cu,Ni) phases in the joint, where smaller Al_2_(Cu,Ni) phases enhance the compatibility of deformation in the base material [34], reduce stress concentration, and improve the post–fracture elongation of the brazed joint.

The fracture morphology of the Al–10Si–10Cu–5Zn–2.0Ni brazed joint exhibits a uniform distribution of tear ridges and dimples, resulting in good mechanical properties. The tensile strength serves as the criterion for evaluating the quality of the brazing. Y element is added for modification based on the Al–10Si–10Cu–5Zn–2.0Ni alloy. The fracture morphology is shown in Figure 14e–g. As seen in the figures, when the Y content is 0.4 wt.%, the distribution of tear ridges and dimples is relatively uniform, which contributes to an increase in the tensile strength of the brazed joint.

## 5. Conclusions

In this study, Al–10Si–10Cu–5Zn–*x*Ni–*y*Y filler alloys were designed and fabricated, and their mechanical properties were investigated using first–principles calculations. The melting behavior, microstructure, and brazed joint performance of the filler alloys were systematically studied. The principal findings of this study are summarized as follows:

(1) The Al–10Si–10Cu–5Zn–2.0Ni–0.4Y filler alloy exhibits high strength and hardness (E = 140.48 GPa, G = 53.64 GPa) while also possessing good toughness (Cauchy pressure = 33.29 GPa, ν= 0.310), thereby meeting the mechanical performance requirements for the brazed joint.
(2) The Al–10Si–10Cu–5Zn–2.0Ni–0.4Y filler alloy is mainly composed of α–Al solid solution, Si phase, Al_2_Cu phase, and Al_2_(Cu,Ni) intermetallic compounds. The addition of Ni promotes the formation of fine Al_2_(Cu,Ni) phases, while the introduction of Y further refines the grain structure, enhances wettability, and reduces interfacial porosity. However, when the Y content exceeds 0.4 wt.%, excessive modification occurs, deteriorating the microstructure of the filler alloy. Therefore, the optimal Y content range in this study is 0–0.4 wt.%.
(3) Brazing of 7072 aluminum alloy using Al–10Si–10Cu–5Zn–2.0Ni and Al–10Si–10Cu–5Zn–2.0Ni–0.4Y fillers produced joints with excellent tensile strength, demonstrating that Ni and Y effectively enhance joint strength. The consistency between computational and experimental results confirms that first–principles calculations provide an effective approach for predicting the performance of aluminum–based filler alloys.
(4) The outcomes of this research serve as a theoretical basis for the compositional design of Al–Si–Cu–based brazing materials used in industry, with potential applications in automotive heat exchangers and lightweight structural components.
(5) Future research should integrate in situ experiments with computational modeling to elucidate the roles of Ni and Y in the interfacial reaction layer and to evaluate their stability under high–temperature service conditions.

## Figures and Tables

**Table 1:** Chemical Composition of 7072 Aluminum Alloy Base Material (wt.%).

Grades	Chemical Composition/wt.%									
	Si	Fe	Cu	Mn	Mg	Cr	Zn	Zr	Ti	Al
7072	0.35	0.4	0.2	0.05~0.5	1.0~1.4	0.1~0.35	4.0~5.0	0.08~0.2	0.05	Bal.

**Table 2:** Chemical Composition of Al–10Si–10Cu–5Zn–*x*Ni–*y*Y Filler Alloy (wt.%).

Solder	Si	Cu	Zn	Ni	Y	Al
1#	10	10	5	—	—	Bal.
2#	10	10	5	1.0	—	Bal.
3#	10	10	5	2.0	—	Bal.
4#	10	10	5	3.0	—	Bal.
5#	10	10	5	2.0	0.2	Bal.
6#	10	10	5	2.0	0.4	Bal.
7#	10	10	5	2.0	0.6	Bal.

**Table 3:** Elastic constants (Cij), bulk modulus (B), shear modulus (G), Young’s modulus (E), Cauchy pressure, B/G ratio and Poisson’s ratio (ν) of the Al–10Si–10Cu–5Zn–*x*Ni–*y*Y alloy.

Compounds	C_11_	C_12_	C_44_	B	E	G	C_12_–C_44_	B/G	ν
	These Units Are All Gpa								
x = 0, y = 0	230.6	41.57	15.60	104.58	95.18	35.29	25.97	2.963	0.348
x = 1, y = 0	211.79	51.31	22.63	104.81	103.39	38.71	28.68	2.710	0.336
x = 2, y = 0	210.26	55.12	24.02	106.83	105.05	39.30	30.40	2.720	0.336
x = 3, y = 0	203.14	60.61	25.40	108.12	104.38	39.98	35.21	2.774	0.339
x = 2, y = 0.2	200.11	80.14	48.60	120.13	138.33	52.88	31.53	2.272	0.308
x = 2, y = 0.4	188.37	90.19	56.91	122.92	140.48	53.64	33.29	2.292	0.310
x = 2, y = 0.6	161.06	110.14	79.71	127.11	133.84	50.52	30.43	2.516	0.325

**Table 4:** Experimentally measured melting points of Al–10Si–10Cu–5Zn–*x*Ni–*y*Y alloys.

Filler Alloy	T_onset_ (°C)	T_offset_ (°C)	T_m_ (°C)	ΔT (°C)
Al–10Si–10Cu–5Zn	506.592	566.587	556.384	49.792
Al–10Si–10Cu–5Zn–1.0Ni	505.825	575.826	559.372	53.547
Al–10Si–10Cu–5Zn–2.0Ni	505.825	565.946	550.696	44.871
Al–10Si–10Cu–5Zn–3.0Ni	505.825	560.747	542.893	37.068
Al–10Si–10Cu–5Zn–2.0Ni–0.2Y	505.806	565.091	549.06	43.254
Al–10Si–10Cu–5Zn–2.0Ni–0.4Y	505.806	565.083	547.891	42.085
Al–10Si–10Cu–5Zn–2.0Ni–0.6Y	505.806	565.441	545.765	39.959

**Table 5:** Al_2_(Cu,Ni) phase grain size in Al–10Si–10Cu–5Zn–*x*Ni–*y*Y filled alloys.

Filler Alloy	Minimum Grain Size/μm	Maximum Grain Size/μm	Average Grain Size/μm	Area Fraction/%
Al–10Si–10Cu–5Zn	2.15	39.31	5.21	10.3
Al–10Si–10Cu–5Zn–1.0Ni	1.49	38.49	4.69	8.12
Al–10Si–10Cu–5Zn–2.0Ni	1.39	36.33	3.82	7.9
Al–10Si–10Cu–5Zn–3.0Ni	1.26	45.24	5.13	8.78
Al–10Si–10Cu–5Zn–2.0Ni–0.2Y	1.24	25.70	2.94	7.29
Al–10Si–10Cu–5Zn–2.0Ni–0.4Y	1.17	22.02	2.58	6.98
Al–10Si–10Cu–5Zn–2.0Ni–0.6Y	1.02	16.86	1.83	6.64

**Table 6:** Chemical composition (wt.%) and possible phases of the labeled regions in Figure 10.

Region	Al	Si	Cu	Zn	Ni	Y	Mn	Fe	Possible Phase
A	99.04	–	–	0.96	–	–	–	–	α–Al
B	23.46	76.54	–	–	–	–	–	–	Si
C	68.3	1.81	29.89	–	–	–	–	–	Al_2_Cu
D	–	99.8	–	0.2	–	–	–	–	Si
E	66.49	–	32.79	–	0.71	–	–	–	Al_2_(Cu,Ni)
F	91.75	–	–	–	–	–	1.51	6.74	Al_6_(Mn,Fe)
G	64.09	–	33.86	2.05	–	–	–	–	Al_2_Cu
H	66.09	–	33.18		0.73	–	–	–	Al_2_(Cu,Ni)
I	83.41	5.79	–	1.65	–	–	2.1	7.05	Al_8_(Mn,Fe)_2_Si
J	28.97	70.27	–	–	–	–	–	–	Si
K	58.67	–	21.12	0.98	18.32	0.91	–	–	Al_2_(Cu,Ni)

**Table 7:** Mechanical properties of Al–10Si–10Cu–5Zn–*x*Ni–*y*Y brazed joints.

Filler Alloy	Tensile Strength/Mpa	Elongation After Break/%	Microhardness/HV_0.2_
Al–10Si–10Cu–5Zn	250	9.31	173.35
Al–10Si–10Cu–5Zn–1.0Ni	256.9	5.77	178.88
Al–10Si–10Cu–5Zn–2.0Ni	275.3	6.96	185.28
Al–10Si–10Cu–5Zn–3.0Ni	267.5	8.35	185.56
Al–10Si–10Cu–5Zn–2.0Ni–0.2Y	285.4	4.05	190.8
Al–10Si–10Cu–5Zn–2.0Ni–0.4Y	295.1	4.76	193.48
Al–10Si–10Cu–5Zn–2.0Ni–0.6Y	280.8	5.05	195.64

**Table 8:** EDS analysis results of typical phases of fracture morphology of Al–10Si–10Cu–5Zn–*x*Ni–*y*Y brazed joints.

Region	Al	Si	Cu	Zn	Ni	Y	Possible Phase
A	5.02	91.58	2.61	0.79	–	–	Si
B	47.89	0.53	48.24	3.34	–	–	Al_2_Cu
C	61.34	1.79	27.03	1.26	8.58	–	Al_2_(Cu,Ni)

## Data Availability

The original contributions presented in this study are included in the article. Further inquiries can be directed to the corresponding authors.
